# Questionnaire dataset: Attitude of epileptologists and obstetricians to pregnancy among women with epilepsy

**DOI:** 10.1016/j.dib.2020.105948

**Published:** 2020-06-29

**Authors:** Makiko Egawa, Keiko Hara, Masami Ikeda, Masayuki Yoshida

**Affiliations:** a Department of Nutrition and Metabolism in Cardiovascular Disease, Graduate School of Medical and Dental Sciences, Tokyo Medical and Dental University, 1-5-45 Yushima, Tokyo, Bunkyo-ku 113-8519, Japan; b Department of Respiratory and Nervous System Science, Graduate School of Medical and Dental Sciences, Tokyo Medical and Dental University, Japan; c Hara Clinic, Yokohama, Japan; d Faculty of Education and Humanities, Department of Psychology, Jumonji University, Japan; e Department of Life Science and Bioethics, Graduate School of Medicine, Tokyo Medical and Dental University, Japan

**Keywords:** Epilepsy, Pregnancy, Obstetrician, Coordination, Women with epilepsy, Counseling

## Abstract

This data article presents supplementary material for our report “Role of obstetricians in promoting pregnancy-related knowledge among women with epilepsy in Japan” (Egawa et al. 2020). To provide more effective preconception counseling and perinatal care, we surveyed the attitude of epileptologists and obstetricians about pregnancy-related issues in women with epilepsy (WWE). Obstetricians said they needed information from epileptologists about seizure for management of WWE. Half of epileptologists did not feel the need to communicate with obstetricians before their patients’ pregnancy; they only contacted obstetricians after their patients became pregnant, and they only referred patients to an obstetrician if requested by the patient.

Specifications Table

**Table:** 

**Subject**	Obstetrics, Gynecology and Women's Health
**Specific subject area**	Management of pregnant women with medical complications
**Type of data**	Tables
	Figures
**How data were acquired**	Questionnaire survey
**Data format**	Raw, Analyzed
**Parameters for data collection**	Not applicable
**Description of data collection**	We selected 400 board-certified epileptologists and 300 board-certified obstetricians nationwide from a list of websites for each speciality's scientific society.
**Data source location**	1–5–45 Yushima, Bunkyo-ku, Tokyo, Japan
**Data accessibility**	Data are available within this article
**Related research article**	M. Egawa, K. Hara, M. Ikeda, E. Kono, S. Miyashita, N. Miyasaka, M. Inaji, T. Maehara, M. Yoshida. Role of obstetricians in promoting pregnancy-related knowledge among women with epilepsy in Japan, Epilepsy Behav. In Press.

**Value of the Data**

• This dataset provides the viewpoints of epileptologists and obstetricians toward pregnancy-related issues of WWE.
• These data help readers to understand present issues and the need to build a system of coordination between epileptologists and obstetricians.
• These data, obtained from epileptologists and obstetricians, provide additional insight to the accompanying article [1], which only includes information from WWE.

## 1 Data description

The data in this article consist of additional figures and tables provided in a report [1]. Figure 1 shows the background of respondents to our questionnaire. Figure 1–1 shows years of experience, Figure 1–2 shows the type of workplace, and Figure 1–3 shows the subspecialities of epileptologists. Half of epileptologists were pediatricians. Table 1 shows obstetricians’ responses regarding information required for the management of perinatal care in WWE. Many obstetricians were concerned about the status of seizure and wanted to know how to handle seizure to manage perinatal care in WWE. Obstetricians also wanted to know how to contact epileptologists if needed. Table 2 shows epileptologists’ responses regarding cooperation with obstetricians. Half of epileptologists did not feel the need to communicate with obstetricians before their patients’ pregnancy (Table 2–1); most epileptologists contacted obstetricians after their patients became pregnant or if requested by the patient (Table 2–2). Table 2–3 shows how epileptologists refer their patients to obstetricians. Only 26.9% of epileptologists had an affiliated maternity unit.

## 2 Experimental design, materials and methods

We mailed questionnaires and consent forms. In total, 115 epileptologists returned the questionnaires (response rate, 29%) and 187 obstetricians returned the survey (response rate, 62%). Data were collected from May to June 2019.

## 3 Ethics statement

Informed consent was obtained from respondents, and all procedures performed in the study were in accordance with the principals of the Declaration of Helsinki. This study was approved by the Medical Research Ethics Committee of Tokyo Medical and Dental University (M2018–290).

## Declaration of Competing Interest

The authors declare that they have no known competing financial interests or personal relationships which have, or could be perceived to have, influenced the work reported in this article.

## Appendix Supplementary materials

Image, application 1

Image, application 2

Image, application 3
