# Supplementary material for: Questionnaire dataset: Attitude of epileptologists and obstetricians to pregnancy among women with epilepsy
Source: Data Brief. 2020 Jun 29;31:105948. doi: 10.1016/j.dib.2020.105948 (PMC7347994; doi:10.1016/j.dib.2020.105948)
Supplement: Supplementary file 1 [file mmc1.zip › Data in Breif data 20200511.pptx]

## Slide 1
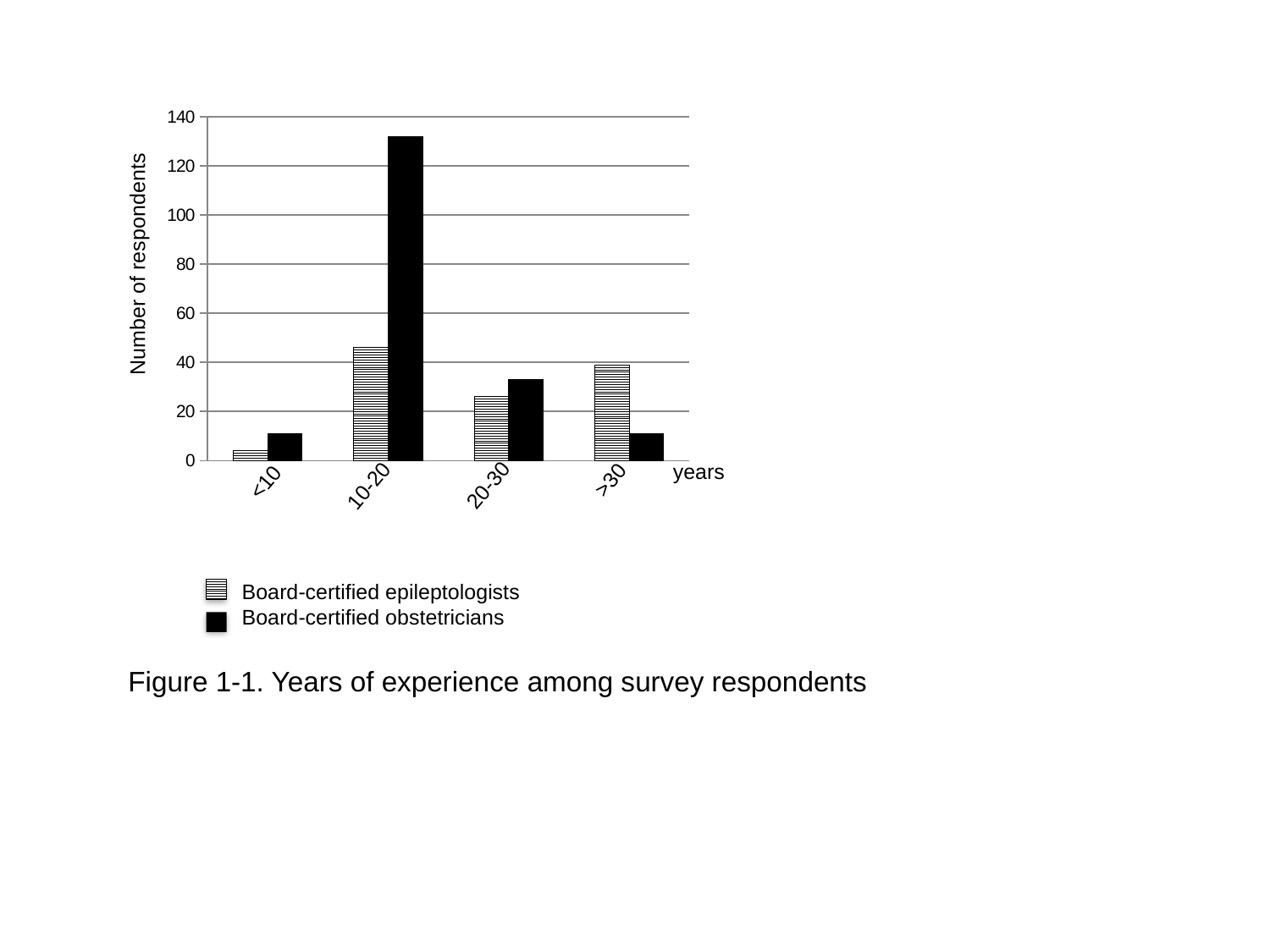

### Chart
| Category | | |
|---|---|---|
| 〜10年 | 4.0 | 11.0 |
| 11〜20年 | 46.0 | 132.0 |
| 21〜30年 | 26.0 | 33.0 |
| 30年〜 | 39.0 | 11.0 |Number of respondents
years
>30
<10
20-30
10-20
Board-certified epileptologists
Board-certified obstetricians
Figure 1-1. Years of experience among survey respondents

## Slide 2
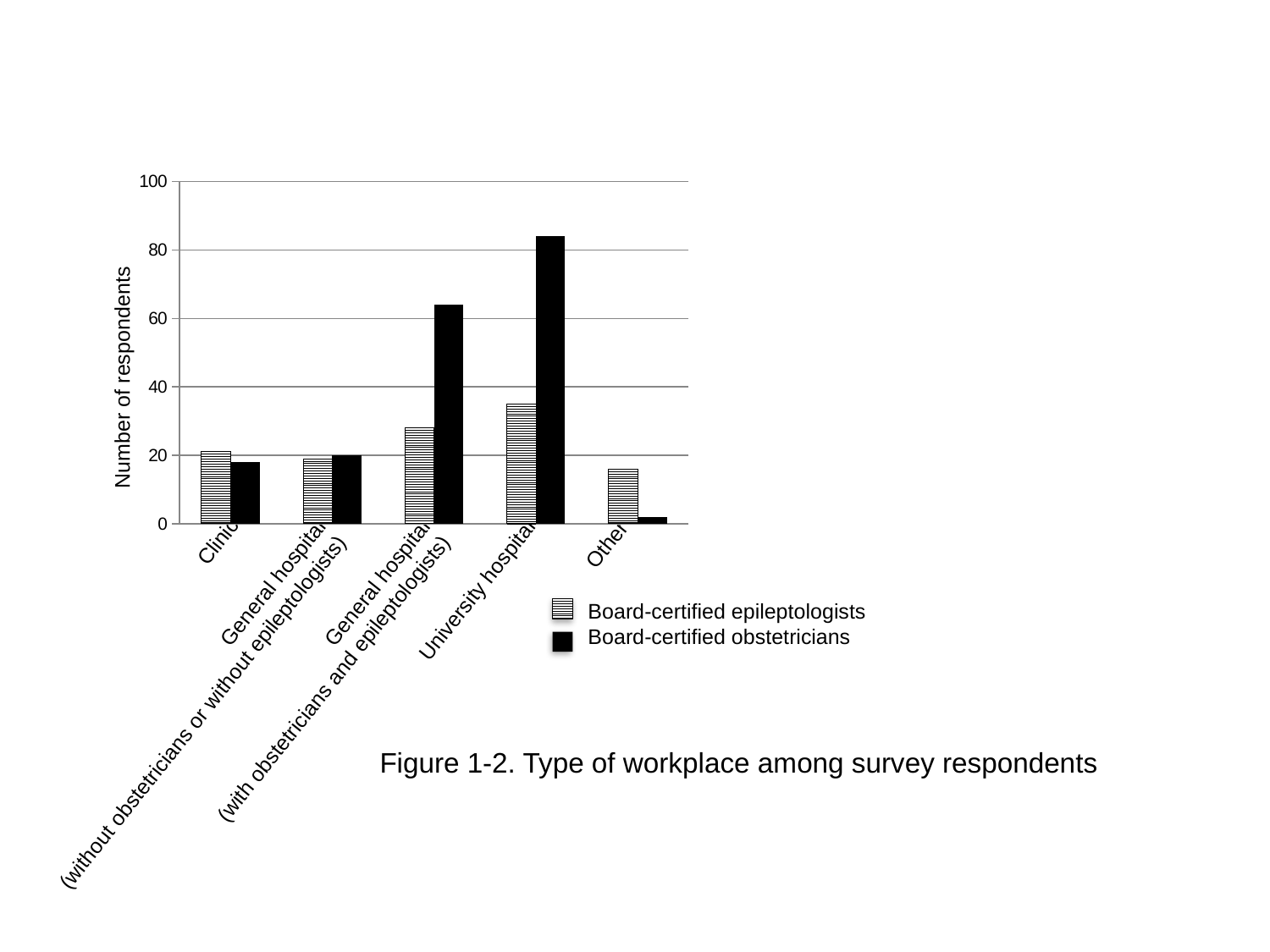

### Chart
| Category | | |
|---|---|---|Number of respondents
Clinic
Other
University hospital
Board-certified epileptologists
Board-certified obstetricians
　　General hospital
(with obstetricians and epileptologists)
　　General hospital
(without obstetricians or without epileptologists)
Figure 1-2. Type of workplace among survey respondents

## Slide 3
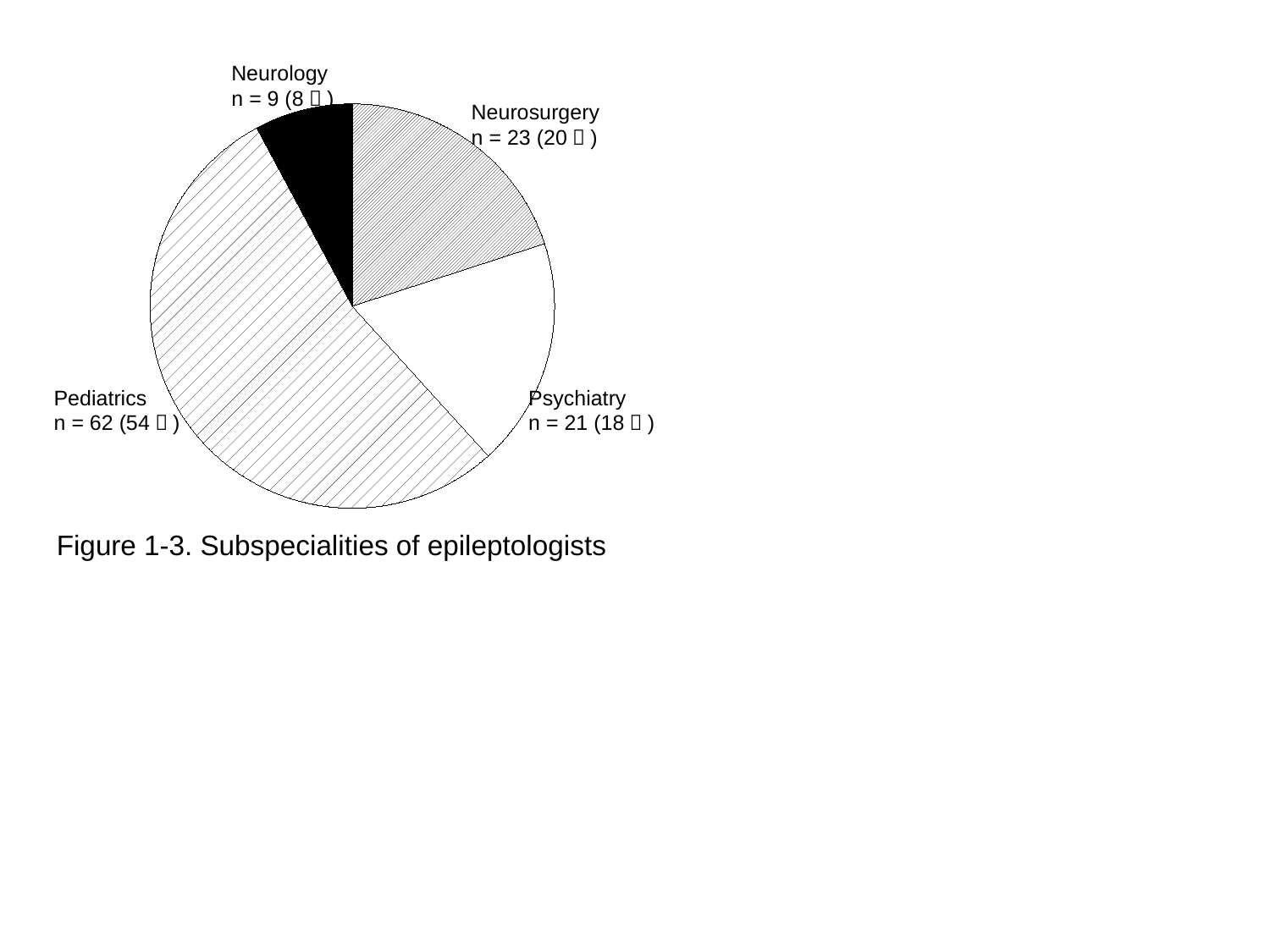

Neurology
n = 9 (8％)
Neurosurgery
n = 23 (20％)
### Chart
| Category | |
|---|---|Pediatrics
n = 62 (54％)
Psychiatry
n = 21 (18％)
Figure 1-3. Subspecialities of epileptologists

## Slide 4
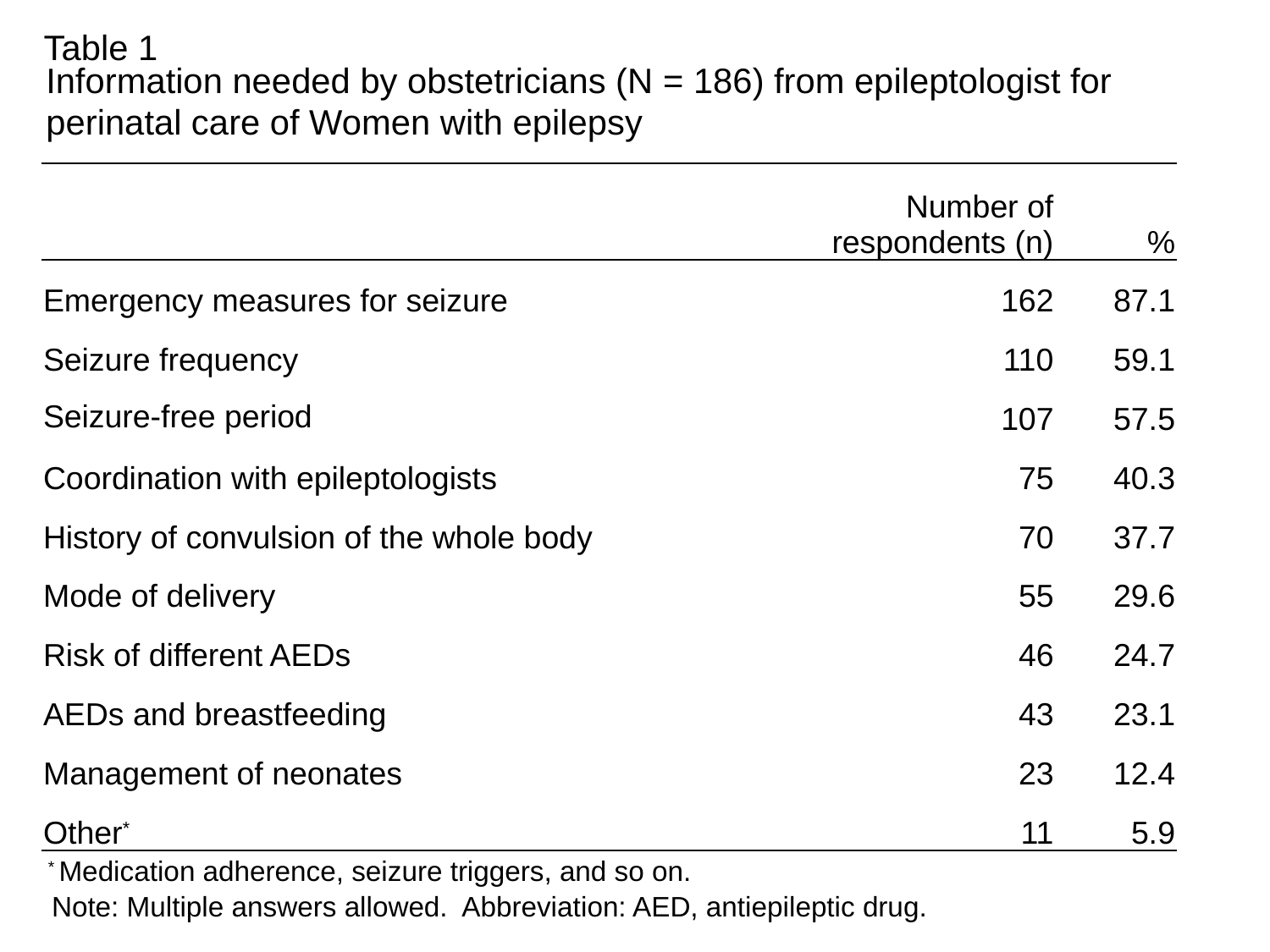

Table 1
Information needed by obstetricians (N = 186) from epileptologist for perinatal care of Women with epilepsy
| | Number of respondents (n) | % |
| --- | --- | --- |
| Emergency measures for seizure | 162 | 87.1 |
| Seizure frequency | 110 | 59.1 |
| Seizure-free period | 107 | 57.5 |
| Coordination with epileptologists | 75 | 40.3 |
| History of convulsion of the whole body | 70 | 37.7 |
| Mode of delivery | 55 | 29.6 |
| Risk of different AEDs | 46 | 24.7 |
| AEDs and breastfeeding | 43 | 23.1 |
| Management of neonates | 23 | 12.4 |
| Other\* | 11 | 5.9 |
* Medication adherence, seizure triggers, and so on.
Note: Multiple answers allowed. Abbreviation: AED, antiepileptic drug.

## Slide 5
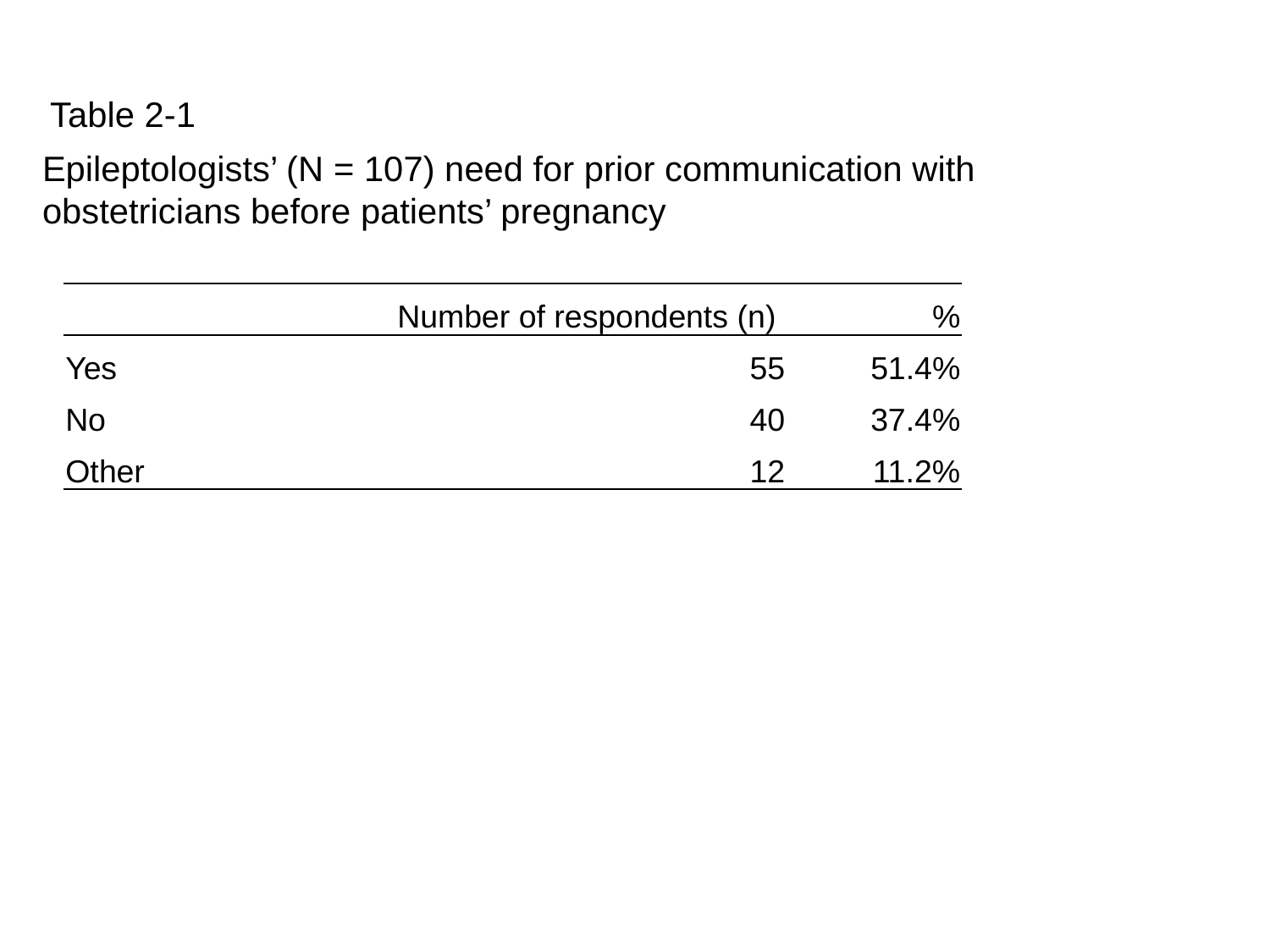

Table 2-1
Epileptologists’ (N = 107) need for prior communication with obstetricians before patients’ pregnancy
| | Number of respondents (n) | % |
| --- | --- | --- |
| Yes | 55 | 51.4% |
| No | 40 | 37.4% |
| Other | 12 | 11.2% |

## Slide 6
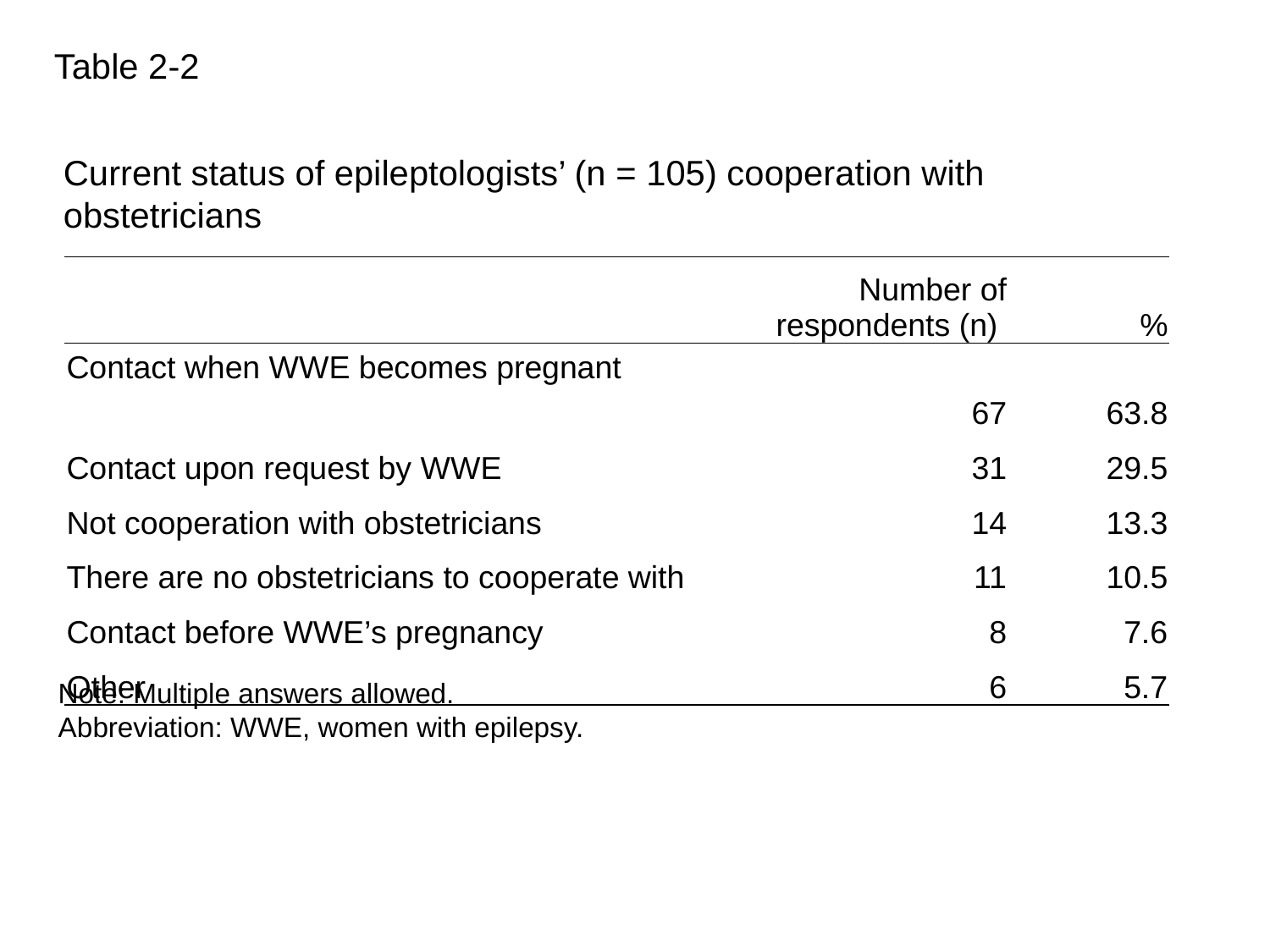

Table 2-2
Current status of epileptologists’ (n = 105) cooperation with obstetricians
| | Number of respondents (n) | % |
| --- | --- | --- |
| Contact when WWE becomes pregnant | 67 | 63.8 |
| Contact upon request by WWE | 31 | 29.5 |
| Not cooperation with obstetricians | 14 | 13.3 |
| There are no obstetricians to cooperate with | 11 | 10.5 |
| Contact before WWE’s pregnancy | 8 | 7.6 |
| Other | 6 | 5.7 |
Note: Multiple answers allowed.
Abbreviation: WWE, women with epilepsy.

## Slide 7
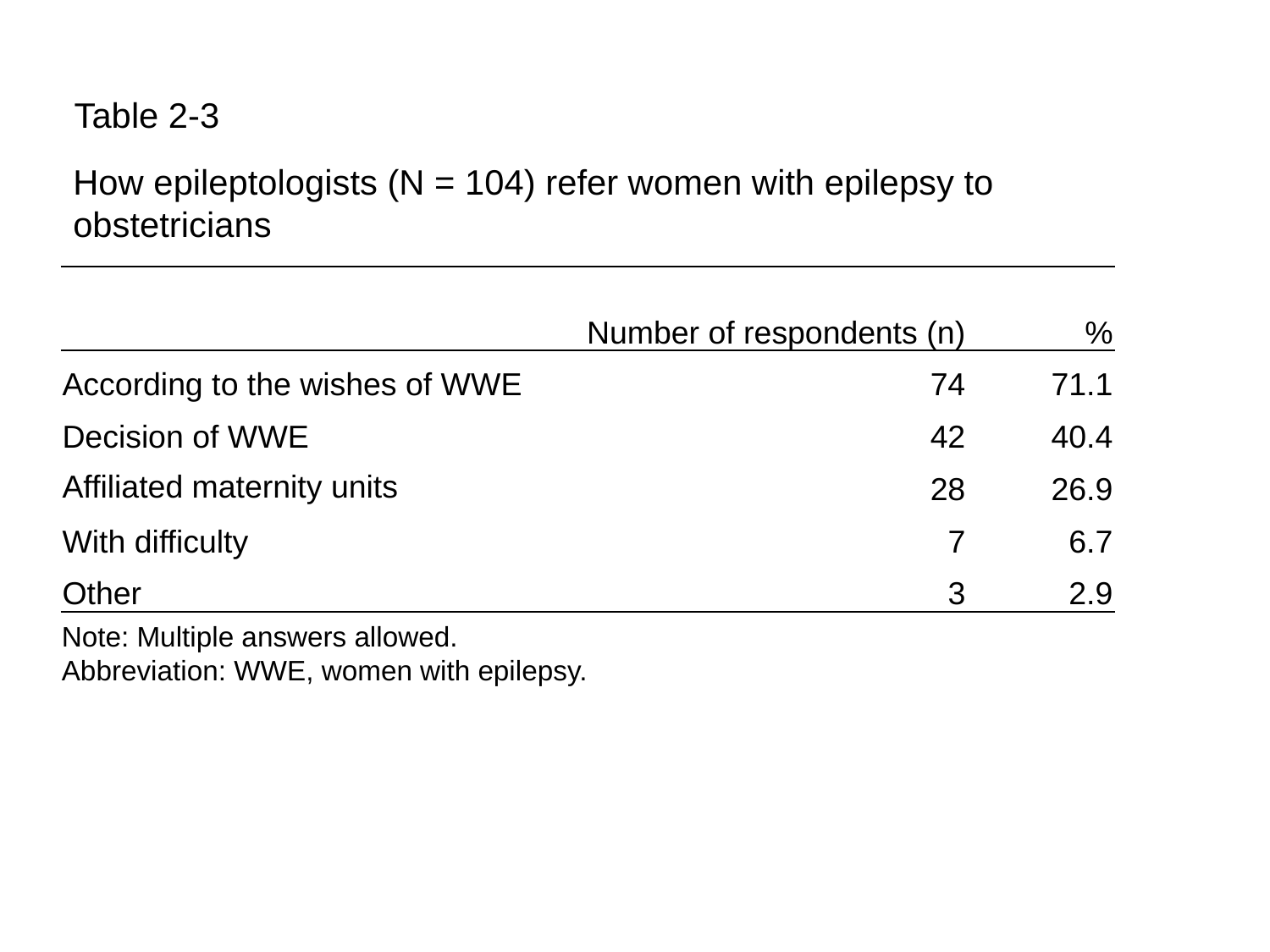

Table 2-3
How epileptologists (N = 104) refer women with epilepsy to obstetricians
| | Number of respondents (n) | % |
| --- | --- | --- |
| According to the wishes of WWE | 74 | 71.1 |
| Decision of WWE | 42 | 40.4 |
| Affiliated maternity units | 28 | 26.9 |
| With difficulty | 7 | 6.7 |
| Other | 3 | 2.9 |
Note: Multiple answers allowed.
Abbreviation: WWE, women with epilepsy.
