# Supplementary material for: Questionnaire dataset: Attitude of epileptologists and obstetricians to pregnancy among women with epilepsy
Source: Data Brief. 2020 Jun 29;31:105948. doi: 10.1016/j.dib.2020.105948 (PMC7347994; doi:10.1016/j.dib.2020.105948)
Supplement: Supplementary file 1 [file mmc1.zip › Epileptologists Questionnaire 20200511.docx]

Questionnaire for board-certified epileptologists

1. Please provide your information.
2. Your years of experience as a physician.
3. Under 10 years
4. 11 to 20 years
5. 21 to 30 years
6. Over 30 years
7. What is your subspeciality?
8. Neurosurgery
9. Psychiatry
10. Pediatrics
11. Neurology
12. Other
13. Describe your workplace.
14. Clinic
15. General hospital without maternity units
16. General hospital with maternity units
17. University hospital
18. Other
19. Please describe your practice regarding WWE
20. Do you provide information about pregnancy-related issues? (Multiple answers allowed)
21. Yes, on a regular basis
22. Yes, upon the patient’s request
23. Yes, upon request from the patient’s parents
24. Yes, upon request from an obstetrician
25. Not particularly
26. Other
27. When do you provide such information? (Multiple answers allowed)
28. Junior high school
29. Senior high school
30. About age 20 years
31. Once the patient has a boyfriend
32. Upon marriage
33. Upon request
34. Upon advise of the parents
35. At the first visit
36. On becoming pregnant
37. Other
38. What is included in such information? (Multiple answers allowed)
39. Risks of AEDs
40. Folic acid supplementation
41. Precautions during pregnancy
42. Mode of delivery
43. AEDs and breastfeeding
44. Child rearing
45. Contraception
46. Inheritance of epilepsy
47. Other
48. Please indicate satisfaction level of patient with your information.

(1= very dissatisfied, 7= very satisfied)

1. Do you think the attitude of patients toward pregnancy changes after receiving such information?
2. Yes
3. No
4. Other

The following two questions are only for those who do not provide information about pregnancy-related issues.

1. Do you think it necessary to provide pregnancy-related information to WWE?
2. Yes, it is necessary
3. No, it is not necessary
4. Other
5. Why do you not provide pregnancy-related information to WWE? (Multiple answers allowed)
6. Never asked
7. No obstetricians available
8. Already prescribe AEDs that have a reduced risk of congenital malformations
9. Never asked by obstetricians
10. Patients have not requested information
11. Other
12. Communication with obstetricians
13. Is it necessary to have prior communication with obstetricians before your patients’ pregnancy?
14. Yes
15. No
16. Other
17. What is the current status of cooperation with obstetricians? (Multiple answers allowed)
18. Contact before WWE become pregnant
19. Contact when WWE become pregnant
20. There are no obstetricians to cooperate with
21. I do not cooperate with obstetricians
22. Contact upon request by WWE
23. Other
24. How to you refer WWE to obstetricians? (Multiple answers allowed)
25. Affiliated maternity units
26. According to the wishes of WWE
27. On the decision of the WWE
28. With difficulty
29. Other
30. How do you communicate with obstetricians when your patients inform you she is pregnant and has regular check-ups with an obstetrician? (Multiple answers allowed)
31. Write a reference letter
32. Call obstetrician
33. Respond to obstetrician’s request for communication
34. Never communicate
35. Other
36. What kind of information do you anticipate from obstetricians after patients’ delivery? (Multiple answers allowed)
37. Weeks of delivery
38. Mode of delivery
39. Birth weight
40. Apgar score
41. Baby’s condition
42. Other
