# Supplementary material for: Questionnaire dataset: Attitude of epileptologists and obstetricians to pregnancy among women with epilepsy
Source: Data Brief. 2020 Jun 29;31:105948. doi: 10.1016/j.dib.2020.105948 (PMC7347994; doi:10.1016/j.dib.2020.105948)
Supplement: Supplementary file 1 [file mmc1.zip › Obstetricians Questionnaire 20200511.docx]

Questionnaire for board-certified obstetricians

1. Please provide your information.
2. Your years of experience as a physician.
3. Under 10 years
4. 11 to 20 years
5. 21 to 30 years
6. Over 30 years
7. Describe your workplace.
8. Clinic
9. General hospital without units for epilepsy
10. General hospital with units for epilepsy
11. University hospital
12. Other
13. Please describe your practice regarding pregnant WWE.
14. Do you have experience in the prenatal management of WWE?
15. Yes
16. No
17. Refuse to manage WWE, owing to lack of epileptologists
18. Other
19. Do you have any trouble with the perinatal care of WWE? (Multiple answers allowed)
20. No
21. Yes, regarding AEDs
22. Yes, regarding mode of delivery
23. Yes, regarding neonatal care
24. Yes, regarding lactation
25. Yes, about communication with epileptologists
26. Other
27. What information do you need from epileptologists for perinatal care of WWE? (Multiple answers allowed)
28. Seizure-free period
29. Seizure frequency
30. History of convulsion of the whole body
31. Emergency measures for seizure
32. Risk of different AEDs
33. Mode of delivery
34. Management of neonates
35. AEDs and breastfeeding
36. Coordination with epileptologists
37. Other
38. Please describe your counseling regarding pregnancy-related issues for WWE.
39. What do you think concerns WWE about pregnancy and delivery? (Multiple answers allowed)
40. Having epilepsy
41. Having another illness
42. Taking AEDs
43. Having insufficient information
44. Inheritance of epilepsy by children
45. No understanding from the partner or family
46. No concerns
47. Other
48. Do you provide information about pregnancy-related issues to WWE?
49. Yes
50. No
51. No, never asked
52. Other
53. When do you provide such information? (Multiple answers allowed)
54. Junior high school
55. Senior high school
56. About age 20 years
57. Once the patient has a boyfriend
58. Upon marriage
59. On becoming pregnant
60. Other
61. Who requests information from you? (Multiple answers allowed)
62. Epileptologists
63. WWE
64. Parents of WWE
65. Obstetricians
66. Other
67. What is included in such information? (Multiple answers allowed)
68. Risks of AEDs
69. Folic acid supplementation
70. Precautions during pregnancy
71. Mode of delivery
72. AEDs and breastfeeding
73. Child rearing
74. Contraception
75. Inheritance of epilepsy
76. Other
77. Are you involved in the prenatal management of the same WWE?
78. Yes
79. No
80. I didn’t know my patient had become pregnant
81. Other
82. Please indicate satisfaction level of patient with your information.

(1= very dissatisfied, 7= very satisfied)

1. Do you think the attitude of patients toward pregnancy changes after receiving such information?
2. Yes
3. No
4. Other

The following two questions are only for those who do not provide information about pregnancy-related issues.

1. Do you think it necessary to provide pregnancy-related information to WWE?
2. Yes, it is necessary
3. No, it is not necessary
4. Other
5. Why do you not provide pregnancy-related information to WWE? (Multiple answers allowed)
6. Never asked
7. Already prescribe AEDs that have a reduced risk of congenital malformations
8. Never asked by epileptologists
9. Patients have not requested information
10. Other
